# Manipulating the interfacial structure of nanomaterials to achieve a unique combination of strength and ductility

**DOI:** 10.1038/ncomms10802

**Published:** 2016-02-18

**Authors:** Amirhossein Khalajhedayati, Zhiliang Pan, Timothy J. Rupert

**Affiliations:** 1 Department of Chemical Engineering and Materials Science, University of California, Irvine, California 92697, USA; 2 Department of Mechanical and Aerospace Engineering, University of California, 4200 Engineering Gateway, Irvine, California 92697, USA

## Abstract

The control of interfaces in engineered nanostructured materials has met limited success compared with that which has evolved in natural materials, where hierarchical structures with distinct interfacial states are often found. Such interface control could mitigate common limitations of engineering nanomaterials. For example, nanostructured metals exhibit extremely high strength, but this benefit comes at the expense of other important properties like ductility. Here, we report a technique for combining nanostructuring with recent advances capable of tuning interface structure, a complementary materials design strategy that allows for unprecedented property combinations. Copper-based alloys with both grain sizes in the nanometre range and distinct grain boundary structural features are created, using segregating dopants and a processing route that favours the formation of amorphous intergranular films. The mechanical behaviour of these alloys shows that the trade-off between strength and ductility typically observed for metallic materials is successfully avoided here.

## Abstract (web summary)

Nanocrystalline metals often exhibit high strength yet suffer from poor ductility. Here, the authors employ grain boundary engineering to overcome this problem by introducing amorphous intergranular films, which enables superior mechanical properties in copper-zirconium alloys.

## Graphical abstract

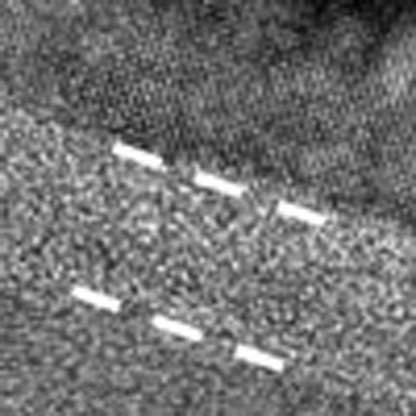

The vast majority of engineering materials are polycrystalline and tailoring the interfaces, or grain boundaries, between the many crystals within a given sample volume offers a promising approach for the discovery of new materials with improved properties. Two recent strategies for realizing such gains have been (i) increasing the number of interfaces or effectively the volume fraction of material located in the interfacial regions and (ii) tailoring the structure of grain boundaries by changing interfacial chemistry. The first strategy is characterized by a dramatic reduction in the size of the crystalline grains that comprise the material and can be termed ‘nanostructuring' when this reduction brings the average grain size below ∼100 nm. The second strategy uses segregating dopant atoms to tailor the local equilibrium structure of interfaces and can be termed ‘complexion engineering'^1^. Although nanostructuring and complexion engineering each offer opportunities on their own, these two material design approaches have not yet been combined in a complementary manner. Natural materials, such as dental enamel^2^ and nacre^3^, often have evolved to contain such hierarchical nanostructures with distinct interfacial features that serve to dramatically influence their performance. Such a strategy applied to engineering materials should allow for the realization of unique property sets, where a material has the benefits of a nanostructured material with regards to one property yet avoids the usual degradation of other properties through complexion engineering.

Nanostructuring has been used extensively in recent years to create a wide range of nanocrystalline metals and ceramics^4^,^5^. These materials take advantage of the fact that atoms in the grain boundaries behave differently than atoms in the grain interior, because of lower packing fractions and the lack of long-range crystalline order. For example, grain boundaries resist the transmission of plastic strain in the form of moving dislocations, as these linear defects in crystals cannot glide through the interfacial region easily. Therefore, a large reduction in grain size is equivalent to adding more obstacles to dislocation motion, leading to a dramatic increase in the material's strength following a well-known scaling law known as Hall–Petch scaling^6^,^7^. Wang *et al.*^8^ reported on nanocrystalline Cu with an average grain size (*d*) of 30 nm and a yield strength of 760 MPa, or more than ten times higher than that of Cu with an average grain size in the micrometre range (55 MPa)^9^. Unfortunately, the addition of a large volume fraction of interfaces can have negative consequences as well. For instance, these same nanocrystalline metals are almost always brittle^9^,^10^, with very little global plastic strain before failure, negating the value of their high strength in many cases.

Separately, it has been recognized that a grain boundary can be described as a type of phase-like structure in many cases, although these interfacial features differ from traditional bulk phases because their structure is dependent on the two abutting crystals and are therefore known as complexions^1^,^11^. However, similar to bulk phases, interface complexions can be analysed with equilibrium thermodynamic theories and may transform between different structures as temperature or chemical composition varies^1^,^12^. Complexions are difficult to observe in materials made from a single element^13^, but recent research has shown that distinct interfacial structures become more accessible in alloys^14^,^15^. Dillon *et al.* created a categorization scheme based on transmission electron microscopy (TEM) observations in doped Al_2_O_3_ that relies on complexion thickness^11^. Six discrete complexion types were found, with increasing levels of structural disorder, which are categorized as (i) single dopant layers, (ii) nominally ‘clean' grain boundaries, (iii) bilayers, (iv) multi-layers, (v) intergranular films with equilibrium thicknesses in the nanometre range and (vi) wetting films with arbitrary thicknesses. Complexion types V and VI are often disordered films without any long-range crystalline order, in which case they can both be classified as amorphous intergranular films (AIFs). An AIF is an example of a complexion that is both distinct in its local structure as well as its effect on material properties, being suggested as the cause for the previously unexplained phenomenon of activated sintering in refractory metals^14^.

To date, complexions have been studied and utilized predominantly in either coarse-grained polycrystalline materials or simple bicrystal samples. Here, we report on a strategy for combining the concepts of nanostructuring and complexion engineering, to enable a new class of materials: nanomaterials with tunable grain boundary structure. We begin by developing a processing route for making such materials, with an eye on techniques that are flexible enough to create a wide variety of chemistries while also scalable so that they can eventually be used to produce bulk quantities of material. Since AIFs are a complexion type that will have atomic structure and properties which are unique compared with normal, clean grain boundaries, we focus on inducing and characterizing these features in a nanoscale grain structure. Then, the mechanical properties of the alloy are probed, to demonstrate the clear advantage of this new materials design strategy. Specifically, we find that the ductility and toughness of nanostructured metals can be greatly improved using complexion engineering without sacrificing any strength, breaking the paradigm of a direct strength-ductility trade-off that has dominated prior observations.

## Results

### Materials selection, processing and characterization

Cu-based alloys were selected for this investigation, as Cu is a common face-centred cubic material and our findings should be broadly applicable to many other structural metals. In addition, the deformation physics and properties of pure nanocrystalline Cu have been well-studied, giving reference points to compare against^16^. As doping of the interfacial regions is required to induce complexions, alloying elements that segregate to the grain boundaries are sought. Empirical considerations such as large atomic size mismatch and limited solubility in the bulk can give rough guidelines for dopant selection, whereas grain boundary segregation enthalpy calculations give a more direct prediction of segregation tendency^17^. As this study is concerned with mechanical behaviour, embrittlement is to be avoided and there is a need to preserve metallic bonding at the interfaces. Non-metals or poor metals such as Bi and Pb are eliminated from consideration. Finally, thermodynamic modelling tools developed by Luo and co-workers^12^,^18^ suggest that AIFs will form if the total energy of an amorphous layer with finite thickness and two new amorphous-crystalline interfaces (that is, the entire complexion) is lower than the energy associated with the interface between two crystalline phases (that is, the original grain boundary). This means that alloy combinations that have a low volumetric free energy penalty for a liquid-like structure and can easily form amorphous solids are promising for the formation of AIFs. With all of these considerations in mind, Zr was chosen as our alloying element, as it will segregate to the grain boundaries^17^, is a transition metal and binary Cu-Zr alloys have a high glass-forming ability^19^.

**Figure 1:**
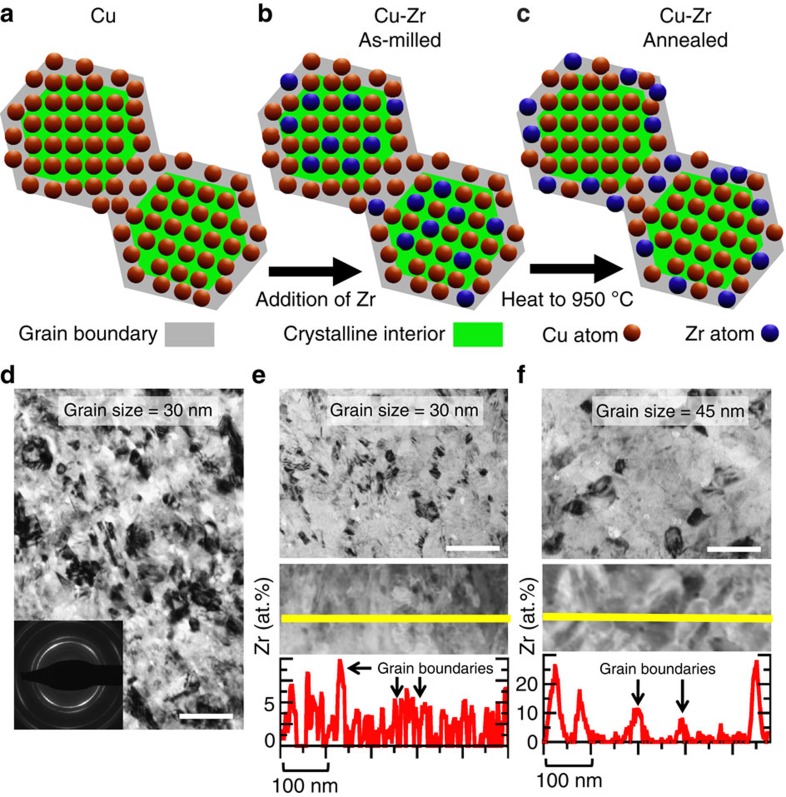
Alloy design strategy for adding segregating dopants. Pure Cu (**a**) can be converted to an alloy by adding Zr during ball milling (**b**). Although the Zr is mixed throughout the grain structure, annealing treatments can then be used to induce preferential Zr segregation to the grain boundaries (**c**). Both the (**d**) pure Cu and (**e**) Cu-Zr as-milled samples have an average grain size of 30 nm (scale bars, 100 nm). (**f**) Annealing the Cu-Zr sample at 950 °C for 1 h allows for segregation and only causes coarsening to a grain size of 45 nm (scale bar, 100 nm). The same level of Zr segregation was found in both the quenched and air-cooled samples.

Mechanical alloying with a high-energy ball mill produces powders with particle sizes of micrometre-scale diameter, with each particle containing many individual nanometre-scale grains. This technique can make nanostructured powders with a wide variety of chemical compositions and scale-up to bulk parts through powder metallurgy techniques is a possibility^20^. As a control, unalloyed nanocrystalline Cu with *d*=30 nm was first produced, as shown in Fig. 1a,d. The selected area electron diffraction pattern in Fig. 1d shows that the material is a random polycrystalline sample. A Cu-3 atomic % Zr alloy was then created, as full segregation of this amount of Zr to the interfaces will put the grain boundary composition into the glass-forming range for Cu-Zr metallic glasses^21^. In the as-milled state, the alloy sample also has *d*=30 nm (Fig. 1e). The bottom frame of Fig. 1e presents a compositional line scan through the as-milled Cu-Zr alloy, showing that Zr is found throughout the sample, mixed into both crystal interior and grain boundary regions (shown schematically in Fig. 1b). Three grain boundaries are labelled in this figure with black arrows. A small amount of grain boundary segregation is occasionally found even in the as-milled sample, as shown by the slightly elevated Zr concentration at the first boundary noted, but significant amounts of Zr were inside of the grains as well. As Zr has negligible solubility (∼0.12 atomic % (ref. 22)) in the Cu lattice according to the bulk phase diagram, this structure is a supersaturated solid solution. To induce segregation, the powders were annealed at 950 °C for 1 h. This temperature is extremely high for Cu and Cu-Zr alloys, being ∼90% of the melting temperature of pure Cu and ∼98% of the solidus temperature (where the material begins to melt) of Cu-3 atomic % Zr^22^. During annealing, Zr diffuses to the grain boundaries, as shown by the compositional line scan in Fig. 1f, where two obvious examples of grain boundaries are labelled. Grain interiors are depleted of Zr after annealing, whereas the interfacial regions show intense Zr enrichment (shown schematically in Fig. 1c). Additional evidence of Zr grain boundary segregation was found by using high-angle annular dark-field TEM imaging (see Supplementary Fig. 1 for details). The Cu-Zr alloy experiences very little grain growth, only coarsening to an average grain size of 45 nm (Fig. 1f), because of a reduction in grain boundary energy with Zr segregation^23^, whereas the grain size of a pure Cu sample annealed under the same conditions coarsens to the micrometre range. Although not the explicit focus of this study, the observed thermal stability at such a high temperature suggests that this material can be consolidated into bulk pieces^20^.

**Figure 2:**
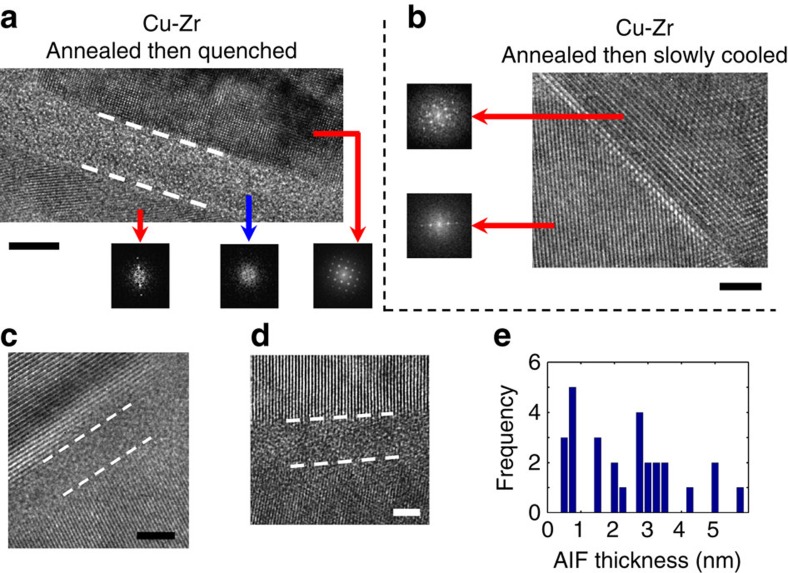
High-resolution TEM images of grain boundary structure in nanocrystalline Cu-Zr alloys. (**a**) An amorphous intergranular film with thickness of 5.7 nm was observed at a grain boundary after quickly quenching from 950 °C (scale bar, 5 nm). (**b**) In contrast, grain boundaries in a slowly cooled sample, with structures that are in equilibrium near ambient temperatures, are all ordered interfaces (scale bar, 2 nm). Insets are fast Fourier transform patterns, highlighting the disordered nature of the interface in **a**. (**c**,**d**) Additional examples of amorphous complexions in the quenched sample (scale bars, 2 nm), whereas **e** summarizes the measurements from the 28 interfacial films found here.

The high-temperature annealing treatment used to induce Zr segregation is also useful for promoting AIF formation. Shi and Luo^12^ recently developed interfacial thermodynamic models and grain boundary diagrams, showing that higher temperatures usually promote thicker AIFs. A set of annealed Cu-Zr powders were quickly quenched by dropping into a large water bath in less than 1 s, freezing in any structures which are in equilibrium at 950 °C. Fresnel fringe imaging^24^ was used to identify interfacial films, followed by high-resolution TEM for detailed characterization of grain boundary structure and measurement of AIF thickness. A representative example of an AIF is presented in Fig. 2a. The areas in the bottom left and top right of Fig. 2a are crystalline, as shown by the presence of lattice fringes in the image as well as sharp spots in the fast Fourier transform patterns, which denote periodic order associated with the lattice. In contrast, the region at the interface, between the two dashed lines, is amorphous and disordered with a thickness of 5.7 nm. The fast Fourier transform pattern shows no sign of long-range crystalline order in this case and is completely featureless. An estimation of the diffusion length for this system shows that these AIFs cannot be a metastable phase, as the high temperature used for annealing provides more than enough kinetic driving force for equilibrium chemical distributions to form within the nanocrystalline grain structure (see Supplementary Note 1 for detailed calculations).

To ensure that the observed AIFs were truly complexions, driven by alloy thermodynamics with a combination of grain boundary dopants and elevated temperatures required, and did not form through another process such as solid-state amorphization, which is purely driven by local composition, another set of powders was annealed at 950 °C and then slowly cooled back to room temperature over a period of ∼5 min, or ∼300 times longer than the quenched sample. Such a cooling schedule gives a sample with interfaces that are in equilibrium at ambient temperatures. No AIFs were observed in this specimen and all interfaces studied were ordered, with a representative example shown in Fig. 2b. Crystalline order is present all of the way up to the boundary. Zr dopants were still found to have segregated to the grain boundaries, with compositional line scans appearing nominally identical to the one shown in Fig. 1f. Although the boundaries were still heavily doped with Zr, the lack of AIFs proves that the amorphous structure shown in Fig. 2a is only in equilibrium at high temperature, in line with thermodynamic theories of disordered complexions.

Additional examples of AIFs from the quenched Cu-Zr sample are shown in Fig. 2c,d with thicknesses of 2.2 and 2.7 nm, respectively. Twenty-eight AIFs were studied with high-resolution TEM, with a distribution of measured AIF thicknesses shown in Fig. 2e. In addition, a number of ordered grain boundaries were observed in this sample as well. As a whole, this means that not all grain boundaries have the same structure in the quenched Cu-Zr alloy, but rather there is a distribution of complexion types. Possible explanations for these variations include differences in grain boundary energy, local segregation state or slight differences in local cooling rates during quenching. The existence of amorphous structures below the solidus temperature of the alloy suggests that these features are stable AIFs with thicknesses determined by a combination of local grain boundary chemical composition and high temperature (type V), although some of the thicker AIFs could possibly be wetting films (type VI). Even though the annealing temperature was below the solidus temperature for Cu-3 atomic % Zr, it was above the eutectic temperature, the lowest possible melting temperature over all mixing ratios, which occurs at higher Zr concentrations. It is possible that some boundaries have higher levels of Zr segregation and move into a different region of the phase diagram where wetting films can form at 950 °C. However, previous observations of complexions in Al_2_O_3_ showed that film thickness changes significantly along individual wetting films^25^, whereas each individual boundary studied here showed no measureable variation in thickness. In addition, wetting films are often much thicker (at least 10 nm thick) than the films observed here. Even if there is some uncertainty in the complexion type of the thickest AIFs, the existence of many nanometre-thick amorphous interfacial structures is clear.

### Mechanical behaviour and the effect of complexions

To demonstrate the potential utility of the material described in this report, a nanostructured metal with disordered amorphous grain boundaries, we turn our attention to mechanical behaviour. Nanocrystalline metals have very high strengths, but are generally also very brittle and exhibit little ductility, with elongations or strain-to-failure values of only a few percent typically reported^9^,^10^. This brittle nature often restricts the usage of nanocrystalline materials in technological applications. Prior attempts to increase the ductility of nanocrystalline metals, for example, by encouraging mechanically driven grain coarsening^26^ or by inducing superplasticity due to sulfur contamination^27^,^28^ or inclusion of a phase with low melting temperature^29^, have come with dramatic reductions in strength. The high strength of a nanocrystalline material comes from the fact that traditional dislocation plasticity mechanisms are shut off at these extremely fine crystallite sizes, requiring dislocations to be nucleated at an interface, propagate across the nanograin and then be absorbed at the opposite grain boundary. The repeated dislocation absorption required for such a physical mechanism may be the cause of the brittle nature of nanocrystalline metals, as atomistic simulations show that a single absorption event leads to high local stresses at the grain boundary^30^, whereas multiple absorption events result in crack nucleation^31^. Such a hypothesis is supported by *in situ* TEM deformation studies, which show that nanometre size cracks nucleate at grain boundary sites during plastic deformation^32^. As a result, damage tolerant grain boundaries should be the key to designing ductile nanocrystalline metals, making control of grain boundary structure a priority.

**Figure 3:**
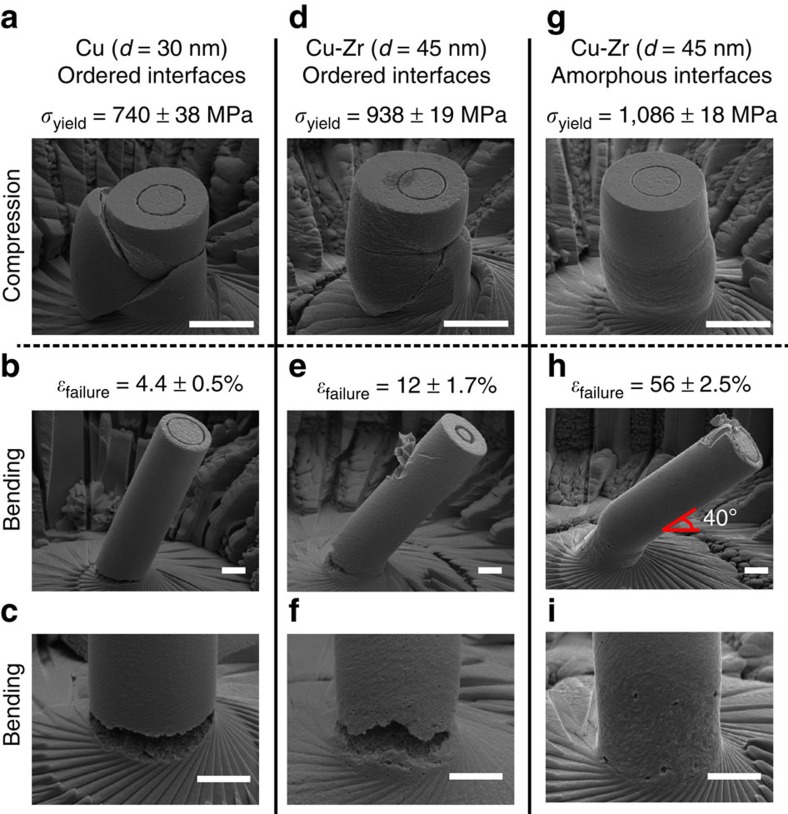
Mechanical testing results from nanocrystalline Cu and Cu-Zr samples. Failed micropillars after compression, as well as yield strength measurements, are shown for (**a**) pure Cu with ordered interfaces, (**d**) Cu-Zr with ordered interfaces and (**g**) Cu-Zr with amorphous intergranular films (scale bars, 5 μm). Failed micropillars after bending, as well as strain-to-failure measurements, are shown for (**b**,**c**) pure Cu with ordered interfaces, (**e**,**f**) Cu-Zr with ordered interfaces and (**h**,**i**) Cu-Zr with amorphous intergranular films (scale bars, 2 μm). A nanocrystalline alloy with AIFs can be both stronger and more ductile than its traditional, pure metal counterpart.

Small cylindrical specimens were created in the powder particles using a focused ion beam microscope, then tested in compression to quantify strength and in bending to quantify strain-to-failure. Representative examples from each test for pure nanocrystalline Cu are shown in Fig. 3a–c, demonstrating the typical response of a nanocrystalline metal to which we can then compare our Cu-Zr results. Figure 3a shows that pure Cu has a high-yield strength of 740 MPa but the pillar cracks and fails during compression, resembling the failure of a brittle ceramic. Compression experiments are not able to quantify the ductility of a material, which is defined as the ability to deform under tensile stress, so pillar bending experiments were used to measure plastic strain-to-failure (see Supplementary Methods for detailed experimental techniques). Videos of representative beam bending experiments for pure Cu and Cu-Zr with AIFs are shown in Supplementary Movies 1 and 2, respectively. Figure 3b,c shows this same brittle response during bending, where a crack forms early during the experiment and there is little plastic strain built up at the base of the pillar, from which strain-to-failure can be measured as 4.4%. This low value is nearly identical to a previous report for nanocrystalline Cu, produced by a different method and tested in uniaxial tension, that had the same average grain size (30 nm) and a similar yield strength (760 MPa)^8^. Representative examples for each test from our Cu-Zr alloy with AIFs are shown in Fig. 3g–i, providing evidence of a strong yet ductile response. Figure 3g shows that the Cu-Zr with AIFs is even stronger than the pure Cu, having a yield strength of 1,086 MPa, but more importantly the pillar compresses in a stable, homogeneous manner. Figure 3h shows that the pillar is ductile during the bending experiment as well and a strain-to-failure of 56% was measured for these alloys, more than ten times larger than the pure Cu value. A magnified view of the pillar base is presented in Fig. 3i, showing that extensive plasticity has occurred and that, even at this large plastic strain, there are only small, unconnected cracks instead of a crack completely across the surface, suggesting that this strain-to-failure value may even be slightly conservative for this alloy. To highlight the importance of AIFs for the observed behaviour, mechanical tests were also performed on the Cu-Zr sample that was slowly cooled in order to have ordered grain boundaries. These results, presented in Fig. 3d–f, demonstrate that this alloy is brittle as well, mimicking the pure Cu sample by crumbling in compression and failing at a small strain in bending. As the only difference between the Cu-Zr samples is the grain boundary structure, with grain size and Zr segregation state identical for both samples, the increased ductility of the Cu-Zr with AIFs can be attributed to the addition of amorphous interfacial films alone.

## Discussion

**Figure 4:**
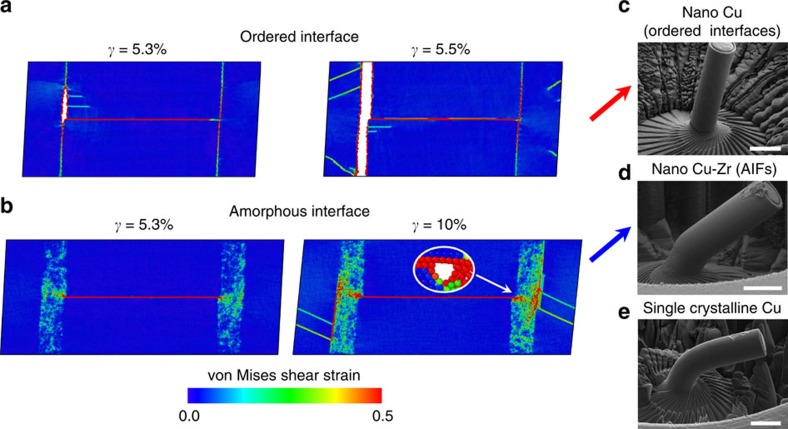
Connection between interfacial structure and damage tolerance. Molecular dynamics simulations of dislocation absorption at (**a**) an ordered grain boundary and (**b**) an amorphous intergranular film show the formation and propagation of crack damage. The ordered interface quickly fractures, whereas the amorphous interface diffuses the strain concentration brought by dislocation absorption and fracture is delayed. The delay of failure explains why (**d**) nanocrystalline Cu-Zr with AIFs has ductility reminiscent of (**e**) coarse-grained Cu while retaining the high strength of (**c**) nanocrystalline Cu (scale bars, 5 μm).

To understand why AIFs act as damage tolerant grain boundaries, molecular dynamics simulations of repeated dislocation absorption at room temperature were carried out on ordered interfaces in both pure Cu and Cu-Zr, as well as Cu-Zr with AIFs. A hybrid Monte Carlo/molecular dynamics simulation technique^33^ was used to create these structures and ensure that they were in equilibrium. For the ordered interfaces in pure Cu, shown in Fig. 4a, very few dislocations can be absorbed before a crack nucleates, leading to cracking at a low applied shear strain (*γ*). The crack then grows quickly after a small additional strain increment until it runs through the specimen. The ordered interfaces in Cu-Zr behaved the same way (see Supplementary Fig. 5 for details). On the other hand, the AIFs diffuse the strain concentration brought by dislocation absorption into a wider region within the boundary (Fig. 4b), delaying the crack nucleation event until a much larger applied shear strain is reached. Pan and Rupert studied the mechanics of this problem systematically in a recent paper, finding that crack nucleation as well as crack growth rate is suppressed as AIF thickness increases^34^. Brandl *et al.* even showed that an AIF can exert attractive forces on nearby dislocations as it is elastically softer than the crystalline lattice, pulling dislocations towards the interface where they can then be absorbed^35^. Experiments on crystalline-amorphous nanocomposite films support this concept as well, as the addition of a thin amorphous layer was found to improve strain-to-failure when compared with films with only a crystalline phase^36^,^37^,^38^. Although the nanocomposites outperform the crystalline films, the strain-to-failure values were only measured to be in the range of 4–14% (much lower than the values reported here), likely because the AIFs were only added in one direction of the film. Donohue *et al.*^39^ reported on another nanolaminate comprised of 90-nm-thick layers of crystalline Cu separated by amorphous Pd-Si layers of 10 nm thickness. Although the monolithic amorphous films had negligible ductility by themselves, the nanolaminate structure had a strain-to-failure of 3%. In contrast, our materials have a fully three-dimensional grain boundary network decorated with AIFs. In our case, by adding amorphous interfacial features, it is possible to have the high strength characteristic of a nanocrystalline metal without the typical brittle failure (Fig. 4c). In fact, the strain-to-failure and plastic flow of a nanocrystalline metal with AIFs (Fig. 4d) resembles that of a coarse-grained or single crystalline metal (Fig. 4e).

**Figure 5:**
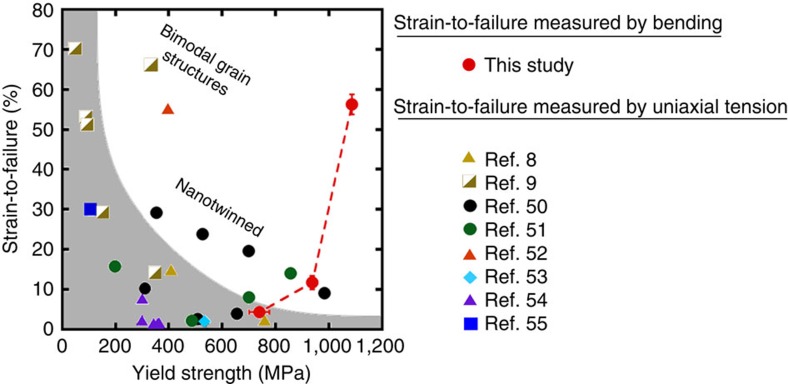
Strain-to-failure and yield strength for Cu and Cu-based alloys. The vast majority of data fall within the grey envelope, with recent advanced alloys pushing slightly outside this limit. Our Cu-Zr alloy breaks the expected trend, with both higher strength and ductility than the pure Cu sample. All strain-to-failure data comes from material that failed under tensile stresses, either under uniaxial tension (literature data) or from the tensile side of a bending experiment (this study). Literature data are taken from refs 8, 9, 50, 51, 52, 53, 54, 55.

The observation of high strength with large ductility fits a recent narrative that nanocrystalline materials are intrinsically ductile on the nanoscale^40^, but suffer from premature failure due to strain localization or cracking^41^. If this premature failure can be suppressed, then the traditional trade-off between strength and ductility can be avoided. One recently developed path for suppression of this premature failure is to incorporate nanocrystalline metals as the outer layer in a gradient architecture, where grain size changes continuously from nanometres to micrometres to avoid early plastic necking^42^,^43^. The results presented here for Cu-Zr alloys created by a unique processing route can be interpreted as another example of this design strategy, with an explicit focus on resisting crack nucleation and growth through the introduction of grain boundary complexions. A strength-ductility synergy is achieved and our Cu-Zr alloys outperform traditional metals. A compilation of data from the literature for Cu and Cu alloys is presented in Fig. 5, where strain-to-failure is plotted as a function of yield strength. The properties of traditional Cu, both microcrystalline and nanocrystalline, fall within the grey envelope, demonstrating a clear trade-off between strength and ductility. Advanced alloys, such as Cu with a bimodal grain structure (micrometre-scale grains embedded in a matrix of nanocrystalline grains) and nanotwinned Cu, push beyond these limits and demonstrate improved combinations of these properties. The Cu and Cu-Zr alloys described in this paper are shown as red points on the plot. It is important to note that our strength measurements come from microcompression tests and strain-to-failure is taken from bending experiments, whereas the data from the literature comes from tensile experiments. The pure Cu sample exhibits classical behaviour and falls within the grey envelope. The strength and strain-to-failure measurements for our pure nanocrystalline Cu are remarkably similar to those of Wang *et al.*^8^, taken from nanocrystalline Cu with an identical grain size created by surface mechanical attrition and appearing as a yellow triangle in Fig. 5. The close agreement between these values demonstrates that it is reasonable to compare our micropillar experiments to the uniaxial tension results available in the literature. Our Cu-Zr with AIFs breaks the traditional paradigm, being both stronger and more ductile than the pure Cu sample. Although the improved ductility has already been discussed, the increased strength comes from the segregation of Zr to the interfaces. Vo *et al.*^44^ used atomistic simulation techniques to study how interfacial segregation altered the grain boundary energy and strength of nanocrystalline Cu. These authors found that boundary energy and strength were intimately related, with strength increasing monotonically as grain boundary energy was reduced by doping. Physically, this means reducing grain boundary energy makes it harder for dislocations to nucleate and then propagate through the grain (the deformation mechanisms, which have been tied to yield strength^45^,^46^). In our case, Zr segregation to grain boundary sites reduces grain boundary energy, which is the inherent driving force for segregation in general but is also supported by the increased thermal stability of Cu-Zr compared with the pure Cu sample. There is even a small increase in strength when going from Cu-Zr with ordered boundaries to Cu-Zr with AIFs. Complexion formation is fundamentally driven by the material seeking a reduction in total interface energy, so this should provide a strengthening contribution. Dislocation nucleation may also be affected by the shift from a crystalline–crystalline interface in the ordered grain boundary to an amorphous–crystalline interface in the case of the AIF. As a whole, the concurrent increase in both strength and ductility shows that control of grain boundary structure is a promising pathway for the design of optimized structural materials.

Our study shows that the previously separate techniques of nanostructuring and complexion engineering can be combined in a complementary manner, introducing a new feature into the design toolbox of materials scientists: grain boundary structure. Unique Cu-Zr alloys are reported, created through powder metallurgy techniques, with AIFs ‘frozen' into the interfaces as a result of Zr segregation and rapid quenching from high-temperature ageing treatments. These new materials demonstrate a combination of strength and ductility that is not found in traditional Cu alloys, behaviour that can be attributed directly to the fracture resistance of AIFs. However, it is important to note that mechanical behaviour is one of many places where the materials described here may be useful. Even if only focusing on nanocrystalline metals with amorphous complexions, we envision materials that can be rapidly consolidated because of faster diffusion along AIFs or that are more tolerant to radiation damage with AIFs acting as sinks for residual point defects^47^. Amorphous interfaces could also act as fast transport paths for energy or electronic applications. We suggest that the introduction of nanocrystalline materials with designed grain boundary structures will dramatically broaden the suite of material properties that can be achieved.

## Methods

### Powder processing and characterization

Nanostructured metals were created by ball milling high-purity powders of the constituent elements with a hardened steel vial and milling media in an argon environment. Powders were then encapsulated in quartz tubes under vacuum and annealed to induce segregation of dopants, either followed by slow air cooling or fast quenching in a water bath. Milling in argon and annealing under vacuum was done to avoid oxidation of the powders. The phase content of the powders was measured using X-ray diffraction, whereas grain size and detailed grain boundary structure were characterized with TEM. The yield strength of each alloy was measured by making micrometre-sized pillars in each powder, followed by compression with an Agilent G200 nanoindenter using a flat triangular diamond tip and an engineering strain rate of 3.1 × 10^-4^ s^−1^. Engineering stress–strain curves were calculated and are presented in Supplementary Fig. 2. Yield strengths were calculated based on a 0.7% plastic strain offset, following the work of Brandstetter *et al.*^48^ Strain-to-failure was calculated from another set of micropillars that were bent with a micro-manipulator inside of a scanning electron microscope. The process for estimating strain-to-failure is illustrated in detail in Supplementary Fig. 3.

### Atomistic modelling

Classical molecular dynamics simulations were performed using the Large-scale Atomic/Molecular Massively Parallel Simulator (LAMMPS) package^49^. The starting configurations for the atomistic simulations were created using a hybrid Monte Carlo/molecular dynamics method capable of finding equilibrium chemical composition and structural configuration. Further details of the processing, mechanical testing and simulation methods can be found in the Supplementary Methods section.

## Additional information

**How to cite this article:** Khalajhedayati, A. *et al.* Manipulating the interfacial structure of nanomaterials to achieve a unique combination of strength and ductility. *Nat. Commun.* 7:10802 doi: 10.1038/ncomms10802 (2016).

## Supplementary Material

Supplementary Information Supplementary Figures 1-6, Supplementary Note 1, Supplementary Methods and Supplementary References

Supplementary Movie 1 In situ bending experiment on pure nanocrystalline Cu. The pillar fractures early in the experiment with little plasticity at the base.

Supplementary Movie 2 In situ bending experiment on nanocrystalline Cu-Zr with AIFs. The pillar is damage resistant and undergoes extensive plasticity at the base without fracturing.
