# Supplementary material for: Manipulating the interfacial structure of nanomaterials to achieve a unique combination of strength and ductility
Source: Nat Commun. 2016 Feb 18;7:10802. doi: 10.1038/ncomms10802 (PMC4759628; doi:10.1038/ncomms10802)
Supplement: Supplementary Information — Supplementary Figures 1-6, Supplementary Note 1, Supplementary Methods and Supplementary References [file ncomms10802-s1.pdf]

Supplementary Figures

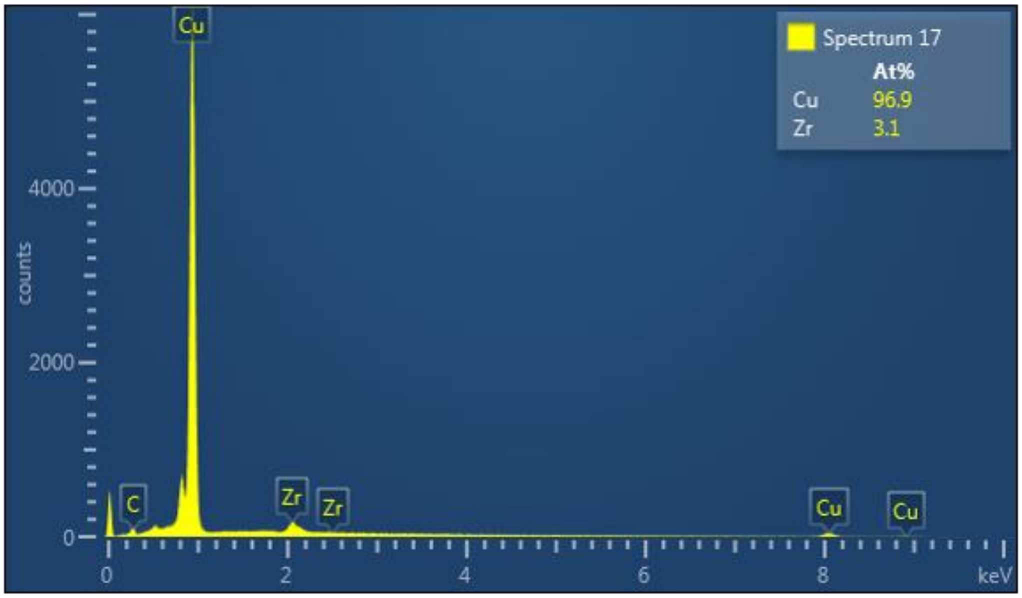

Supplementary Figure 1. EDS spectrum from Cu–3 atomic % Zr powder sample. No contamination from Fe or Cr in the ball milling media is found. Only a small C peak from stearic acid contamination can be observed.

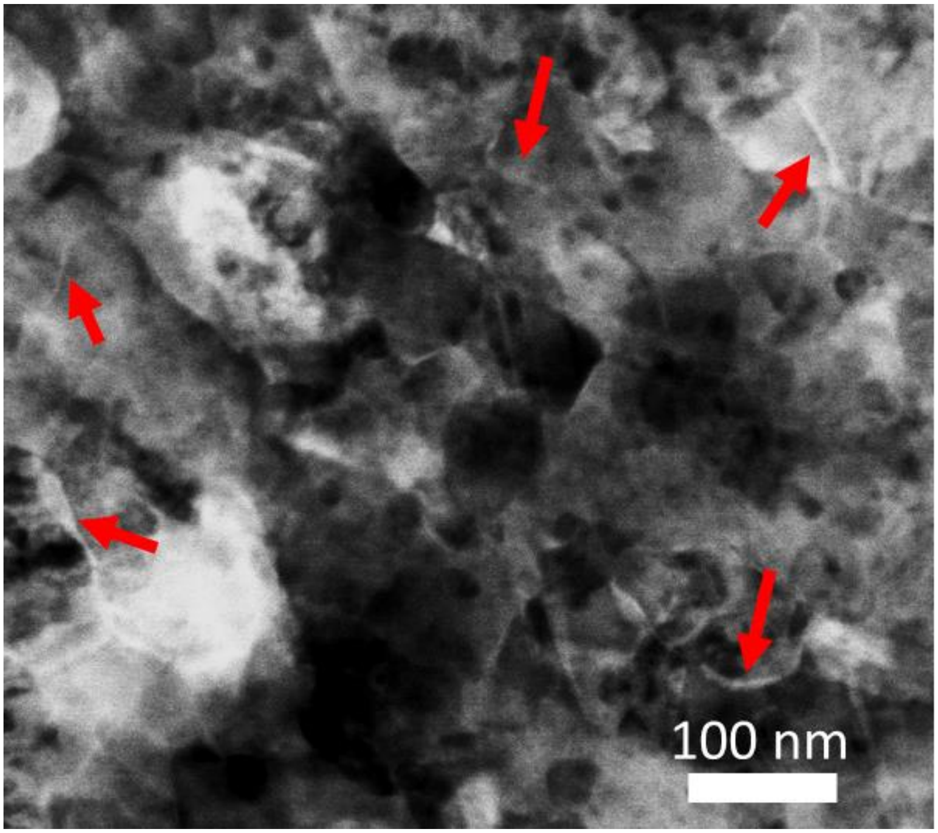

Supplementary Figure 2. HAADF scanning TEM image of nanocrystalline Cu-3 atomic % Zr heat treated to 950 °C and rapidly quenched. Brighter regions have higher concentration of Zr, with a few examples denoted by red arrows showing obvious Zr segregation at the grain boundaries.

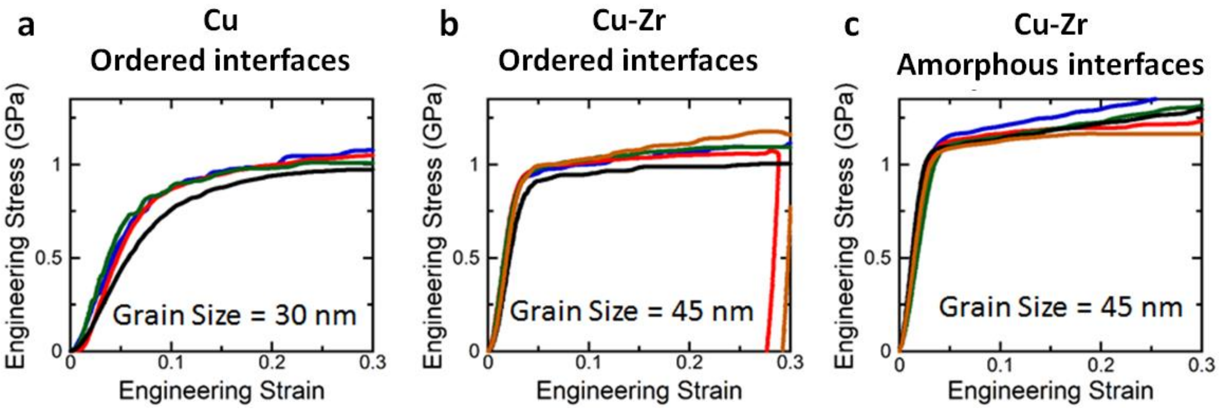

Supplementary Figure 3. Microcompression testing results. Stress-strain curves for (**a**) pure Cu, (**b**) Cu-Zr with ordered grain boundaries, and (**c**) Cu-Zr with amorphous intergranular films. These experiments provide information about the yield strength of each material.

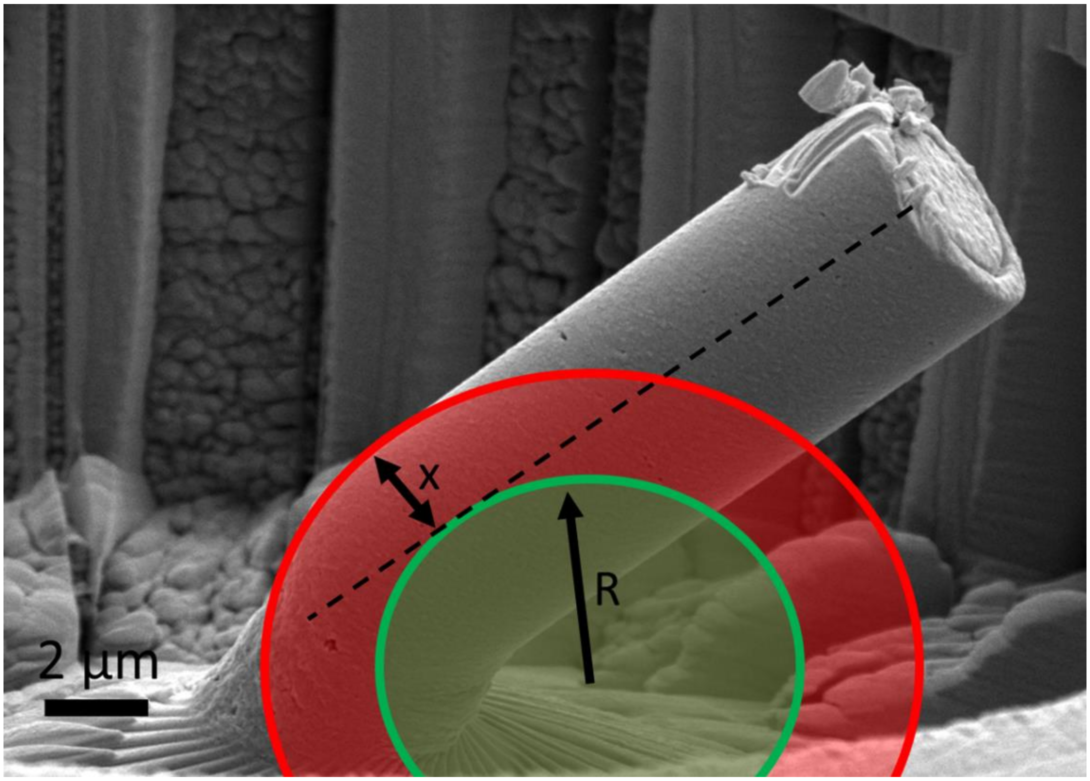

Supplementary Figure 4. Calculation of bending strain after an in-situ bending experiment. The green circle with radius *R* is fitted to the curvature of neutral axis, and the red circle with radius of *R+x* is fitted to the curvature of the edge of the micropillar. The image was taken at a 60° tilt, and the circles are scaled to account for this effect.

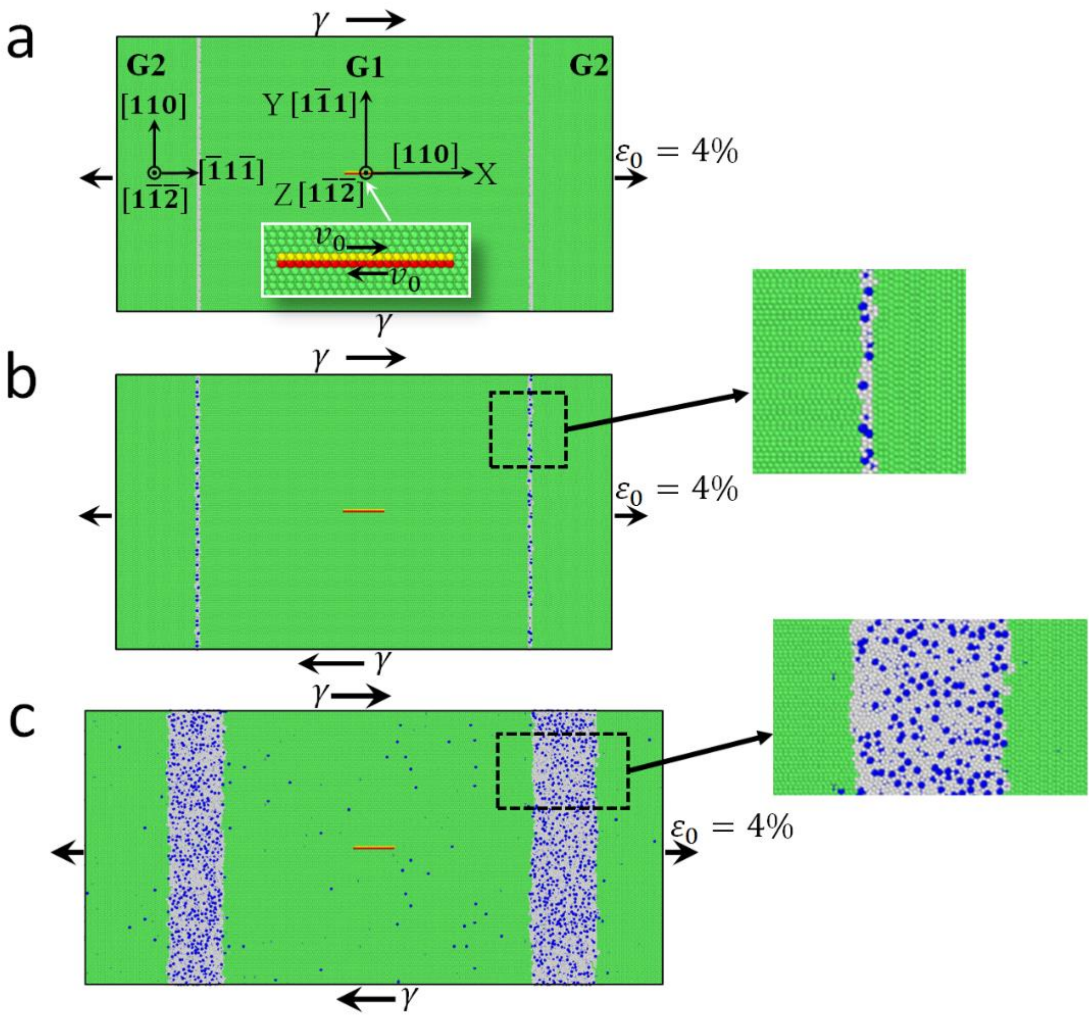

Supplementary Figure 5. Atomistic simulations of dislocation absorption. Bicrystal atomic configurations with (**a**) clean grain boundaries, **(b)** ordered boundaries doped with Zr atoms, and (**c**) amorphous intergranular films. Atoms in grain G1 and G2 are colored green, atoms in grain boundaries or AIFs colored white, and Zr atoms in (**b**) and (**c)** are colored blue. Insets show dislocation sources created in the center grain by moving the yellow and red atoms with respect to each other at a constant speed.

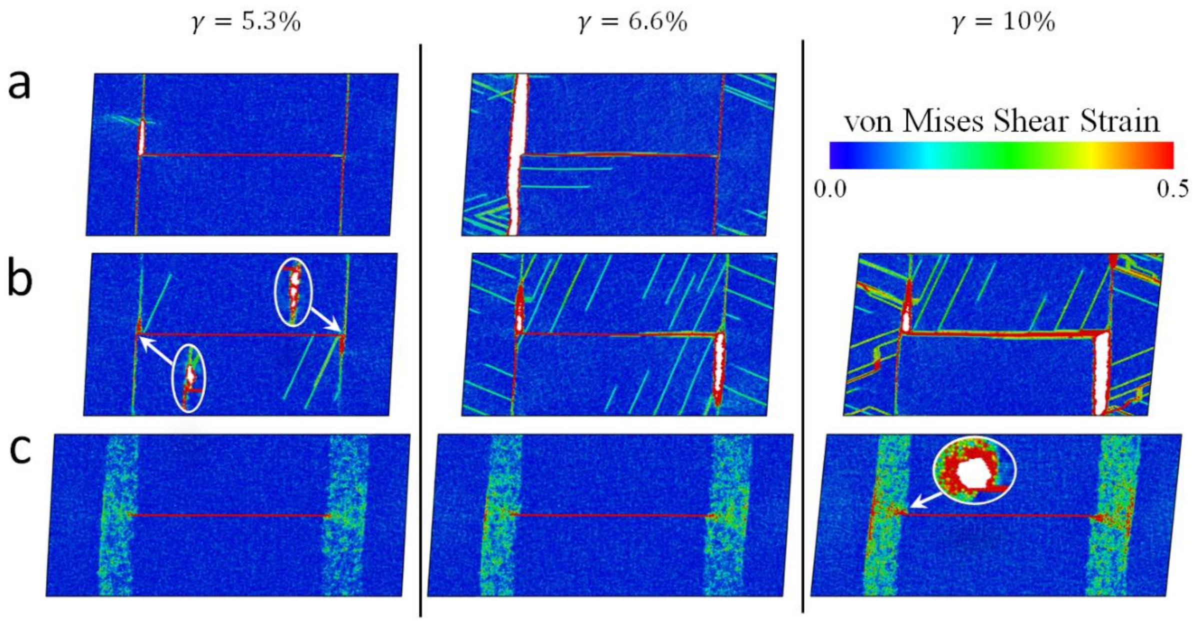

Supplementary Figure 6. The distribution of atomistic von Mises shear strain during dislocation absorption process. Bicrystal atomic configurations with (**a**) clean grain boundaries, **(b)** ordered interfaces doped with Zr atoms, and (**c**) amorphous intergranular films.

Supplementary Note 1

Calculation of Cu-Zr Binary Alloy Interdiffusion at 950 °C

Darken's equation[1] can be used to estimate the Interdiffusion of a binary alloy system:

$$D = X_{Cu} D_{Zr} + X_{Zr} D_{Cu} \quad (1)$$

where D is the interdiffusion coefficient, X_{Cu} and X_{Zr} are the mole fraction of *Cu* and *Zr* in the alloy respectively, and D_{Zr} and D_{Cu} are the effective diffusion coefficients of Zr and Cu. Since the annealing temperature of 950 °C is relatively high for both Zr and Cu (0.56 $T_{\mathrm{melting,Zr}}$ and 0.9 $T_{\mathrm{melting,Cu}}$), we can assume that lattice diffusion coefficient of Cu and Zr as the effective diffusion coefficient for our calculation here. Self-diffusion of pure Cu and Zr can be calculated at 950 °C by using the following equations[2,3]:

$$D_{Cu} = 0.78 \exp(-50.5\, kcal\, mol^{-1}/RT)\ {cm^2}/{s} \quad (2)$$

$$D_{Zr} = 2.4 \times 10^{-4} \exp(-30.1\, kcal\, mol^{-1}/RT)\ {cm^2}/{s} \quad (3)$$

For 950 °C, we calculate D_{Cu} = 7.36 × 10^{-10} cm^2/s and D_{Zr} = 1.00 × 10^{-9} cm^2/s. For our alloy system, X_{Cu} and X_{Zr} are 0.97 and 0.03. With this, we arrive of an interdiffusion coefficient of D = 9.92 × 10^{-10} cm^2/s. The diffusion length can then be calculated as $L = (Dt)^{1/2}$ where time is equal to the 1 hour annealing treatment. The estimated diffusion length for our annealing treatment is then L = 6.24 µm. This

is over 130 times larger than our average grain size, meaning that chemical equilibrium will be achieved.

Supplementary Methods

Materials processing

Nanocrystalline Cu and Cu-3 atomic % Zr powders were produced from -170+400 mesh Cu powder and -50 mesh Zr powder using mechanical alloying. Cu powders with 99.9% purity and Zr powders with 99.7% purity were obtained from Alfa Aesar and Micron Metals, respectively. Mechanical alloying of the powders was performed with a SPEX 8000M mixer/mill equipped with a hardened steel vial and milling media. A ball-to-powder ratio of 10:1 was chosen, and 1 weight % stearic acid was added as a process control agent to minimize cold welding. Since stearic acid can possibly add C, H, and O contamination to the powders, a battery of milling experiments was run to find the minimum amount of process control agent that could be used while still avoiding cold welding of the particles, which causes inefficient grain refinement and mixing. The milling was performed inside of a glove box under a 99.999% pure Ar atmosphere. Both pure and alloyed Cu powders were annealed inside of high purity quartz tubes which were sealed under vacuum at 950 °C inside a tube furnace for 1 h and quenched rapidly in water (within ~1 s). Milling in the glove box and annealing under vacuum were both done to avoid oxidation of the powders. In addition, a control sample from the Cu-Zr alloy was air-cooled to study the effect of cooling rate on the microstructure. The furnace temperature was stable to within ±1 °C, ensuring that the annealing temperature never approached or exceeded the solidus temperature (972 °C). The phase content of the powder samples was measured using a Rigaku Ultima III X-ray diffractometer equipped with a Cu Kα radiation source operating at 40 kV. The powder samples were mounted in a conductive epoxy and mechanically polished to a mirror finish for further characterization.

Electron microscopy and characterization

Transmission electron microscopy (TEM) samples were prepared from individual powder particles using an FEI Quanta 3D dual beam scanning electron microscope (SEM) and focused ion beam (FIB) microscope. The TEM samples were polished with a 5 kV Ga^+ ion beam to remove and minimize any damaged layer from the TEM sample preparation procedure. SEM imaging was performed with the same instrument operating at 5 kV. Energy-dispersive X-ray spectroscopy (EDS) was performed at 20 kV in the SEM/FIB microscope to probe for contamination and chemical inhomogeneity in the sample at the micron scale. The raw EDS spectrum is shown in Supplementary Fig. 1. The EDS detector has a resolution of 0.1 atomic % for transition metals and no Fe or Cr contamination from the steel milling media was detected, meaning that there must be less than 0.1 atomic % Fe or Cr in the powder. EDS is not quantitatively accurate for light elements ($Z < 11$) and in fact cannot measure H at all since it does not release a characteristic X-ray when excited. Even so, no O was detected at all and only a small signal corresponding to C was found. This small level of C contamination, and any H, will be constant for all samples studied in this work, as the amount of stearic acid, or the source of the contamination, was held constant.

TEM bright field images were taken using a FEI/Philips CM-20 TEM operating at 200 kV. The mean grain size for each sample was calculated by manually tracing and measuring the area of at least 100 grains, then calculating the equivalent circular diameters. A Fresnel fringe imaging technique was used to identify grain boundary films, as local differences in atomic density or composition lead to such fringes at interfaces in under- or overfocused conditions[4]. Once interfacial films were found, high resolution TEM was used for thickness measurement, as direct measurements of thickness using Fresnel fringes have been shown to be unreliable[5]. High resolution, phase contrast TEM images were taken using a FEI Titan TEM operating at 300 kV.

Scanning TEM imaging and EDS were performed with the same instrument operating at 80 kV. Supplementary Fig. 2 shows a high angle annular dark field (HAADF) scanning TEM image of the nanocrystalline Cu-3 atomic % Zr annealed at 950 °C and rapidly quenched, showing segregation of Zr to the grain boundaries.

Mechanical testing: Microcompression and bending

At least five micropillars with diameter and length of 5 ± 0.1 μm and 16 ± 0.2 μm, respectively, were fabricated using FIB lathe milling on individual powder particles following the method of Uchic and Dimiduk[6]. This technique produced taper-free pillars so that a uniform stress state can be induced. Since the presence of a pore or cold-welded boundary between two particles near a pillar would dramatically affect the mechanical behavior, care was taken when selecting sites for pillar fabrication. In addition, the pillars were inspected inside of an SEM after fabrication and again after mechanical testing, to ensure that no local porosity was present. Many pillar compression studies have shown that strength increases with decreasing pillar diameter, but this occurs when the sample is small compared to the internal microstructure. Jang et al.[7] and Gu et al.[8] showed that one needs approximately 20-25 grains across the pillar to obtain bulk properties from a microcompression experiment. For a nanostructured material, pillars with micron-scale diameters are actually relatively large. The pillar diameters were >100 times the average grain size, ensuring that the measured response would be representative of "bulk" response and not external size effects. The uniaxial microcompression test was performed with an Agilent G200 nanoindenter using a flat triangular diamond tip and a constant displacement rate of 5 nm/s, giving an engineering strain rate of 3.1×10^{-4} s^{-1}. The load-displacement data was converted to engineering stress-strain using the initial length and diameter of the pillars measured with SEM.

Stress-strain curves for all three samples are presented in Supplementary Fig. 3. Yield strengths, σ_y, were calculated based on a 0.7% plastic strain offset, following the work of Brandstetter et al.[9].

Microbeam bending experiments were performed inside the dual beam FIB/SEM microscope using an Omniprobe micro-manipulator. At least three micropillars were tested in bending by pushing the top of the pillars at a speed of 0.1 µm/s. This testing methodology results in uniaxial strain, ε, at the bottom of the pillars, with tension on one side and compression on the other. The basic geometry of the deformed base resembles a simple beam bending problem, where there is a neutral axis with zero strain and the distance from that neutral axis can be combined with the radius of curvature of the beam to calculate axial strain. To measure the bending strain in each pillar, first we fit a circle with radius, R, to the curvature of the neutral axis and then we fit another circle, with another radius, $R+x$, to the outside edge of the micropillar. This process is shown in Supplementary Fig. 4. Since the measurements are taken after the loading is removed, relieving any elastic deformation, any strain measured is plastic strain and the plastic strain at failure is calculated as $\varepsilon_{failure} = x / R$. Movies of representative beam bending experiments for pure Cu and Cu-Zr with AIFs are shown in Supplementary Movie 1 and Supplementary Movie 2, respectively.

Molecular dynamics simulations of dislocation absorption

Three bicrystal configurations (Supplementary Fig. 5), one pure Cu with clean grain boundaries, one Cu-Zr with ordered interfaces doped by Zr, and one Cu-Zr with 7.2 nm thick amorphous intergranular films, were used to study boundary-dislocation interactions using molecular dynamics simulations performed with the Large-scale Atomic/Molecular Massively Parallel Simulator (LAMMPS) code[10]. The integration time step for all simulations was 1 fs and periodic boundary conditions were applied in all three directions. An embedded-atom method

potential was used to describe Cu-Cu and Zr-Zr interactions, while a Finnis-Sinclair formulation was used to represent Cu-Zr atomic interactions[11]. The orientation of the center grain (G1) in all configurations was chosen so that the resolved shear stress is maximized on one slip plane and drives edge dislocations propagating to the right and left. The second grain (G2) is oriented such that the resolved shear stress on its slip planes along the slip directions is minimized. Therefore, the incoming dislocations are absorbed at the grain boundary and the incompatibility between the two grains is not relaxed by direct dislocation transmission into G2.

Hybrid atomistic Monte Carlo/molecular dynamics simulations were used to produce Cu-Zr with ordered interfaces and AIFs, as shown in Supplementary Fig. 5b and c, respectively. Two reference configurations of pure Cu resembling the one shown in Supplementary Fig. 5a were first created and equilibrated with a conjugate gradient minimization technique. A Nose-Hoover thermostat/barostat was then used to further relax the two samples for 200 ps under zero pressure at 300 and 1223 K, respectively. Thereafter, doping with Zr solutes was simulated using a Monte Carlo method in a variance-constrained semi-grand canonical ensemble[12] after every 100 molecular dynamics steps, with the target global composition of Zr fixed to 0.05 and 2 atomic% Zr for the ordered and AIF samples, respectively. The chemical potential difference between Zr and Cu atoms was chosen to be 3.2 eV to achieve relatively efficient convergence. The two samples are considered to be equilibrated when the absolute value of the fitted slope of the system potential energy over the last 4000 Monte Carlo steps is less than 0.001 eV/step, since additional Monte Carlo steps lead to no considerable structural changes. A conjugate gradient energy minimization was then used to quickly quench the entire system to its local minimum potential energy state, so that the interfacial structure obtained during the doping process can be preserved.

Finally, at 300 K, a Nose-Hoover thermo/barostat was used to further relax the entire system for 40 ps under zero pressure.

To investigate the damage resistance of an interface subject to dislocation absorption, it is necessary to create a positive hydrostatic stress state to promote crack nucleation and growth[13-16]. This was accomplished here by applying an elastic uniaxial tensile strain of 4% on the two samples in the X-direction at a strain rate of 10^9 s^{-1}, in a canonical ensemble. After the pre-tension step, the samples were equilibrated for 200 ps using the canonical ensemble. Shear deformation under the canonical ensemble was then applied to the two samples at an engineering shear strain rate of 10^9 s^{-1} at 300 K. At the same time, an artificial dislocation source in the center of each sample was operated by gradually displacing two layers of atoms with respect to each other at a constant speed to generate dislocation pairs. During the shear deformation, one layer of atoms at the bottom of the samples are held fixed in the vertical direction to prevent rigid body grain rotation. All structural analysis and visualization of atomic configurations was performed using the open-source visualization tool OVITO[17]. The atomic von Mises shear strain of the three samples during shear deformation was presented in Supplementary Fig. 6. Both of the ordered boundaries crack early in the simulation. The added Zr does not have a clear effect on crack nucleation, but it does appear to affect crack growth. The AIF sample on the other hand experiences crack nucleation much later, at strains approximately twice as large as the ordered samples.

Supplementary References

1. Chookajorn, T. & Schuh, C. A. Nanoscale segregation behavior and high-temperature stability of nanocrystalline W–20 at.% Ti. *Acta Mater.* **73**, 128-138 (2014).
2. Butrymowicz, D. B., Manning, J. R. & Read, M. E. Diffusion in Copper and Copper Alloys. Part I. Volume and Surface Self-Diffusion in Copper. *J. Phys. Chem. Ref. Data* **2**, 643-656 (1973).
3. Kidson, G. & McGurn, J. Self-Diffusion in Body-Centered Cubic Zirconium. *Can. J. Phys.* **39**, 1146-1157 (1961).
4. Jin, Q., Wilkinson, D. S. & Weatherly, G. C. Determination of Grain-Boundary Film Thickness by the Fresnel Fringe Imaging Technique. *J. Eur. Ceram. Soc.* **18**, 2281-2286 (1998).
5. MacLaren, I. Imaging and thickness measurement of amorphous intergranular films using TEM. *Ultramicroscopy* **99**, 103-113 (2004).
6. Uchic, M. D. & Dimiduk, D. A. A methodology to investigate size scale effects in crystalline plasticity using uniaxial compression testing. *Mater. Sci. Eng. A* **400**, 268-278 (2005).
7. Jang, D. C. & Greer, J. R. Size-induced weakening and grain boundary-assisted deformation in 60 nm grained Ni nanopillars. *Scr. Mater.* **64**, 77-80 (2011).
8. Gu, X. W. *et al.* Size-Dependent Deformation of Nanocrystalline Pt Nanopillars. *Nano Lett.* **12**, 6385-6392 (2012).
9. Brandstetter, S. *et al.* From micro- to macroplasticity. *Adv. Mater.* **18**, 1545-1548 (2006).
10. Plimpton, S. Fast Parallel Algorithms for Short-Range Molecular-Dynamics. *J. Comput. Phys.* **117**, 1-19 (1995).
11. Mendelev, M. I. *et al.* Development of suitable interatomic potentials for simulation of liquid and amorphous Cu–Zr alloys. *Philos. Mag.* **89**, 967-987 (2009).
12. Sadigh, B. *et al.* Scalable parallel Monte Carlo algorithm for atomistic simulations of precipitation in alloys. *Phys. Rev. B* **85** (2012).
13. Bringa, E. M., Traiviratana, S. & Meyers, M. A. Void initiation in fcc metals: Effect of loading orientation and nanocrystalline effects. *Acta Mater.* **58**, 4458-4477 (2010).
14. Rudd, R. E. & Belak, J. F. Void nucleation and associated plasticity in dynamic fracture of polycrystalline copper: an atomistic simulation. *Comput. Mater. Sci.* **24**, 148-153 (2002).
15. Tang, Y., Bringa, E. M. & Meyers, M. A. Ductile tensile failure in metals through initiation and growth of nanosized voids. *Acta Mater.* **60**, 4856-4865 (2012).
16. Pan, Z. & Rupert, T. J. Damage nucleation from repeated dislocation absorption at a grain boundary. *Comput. Mater. Sci.* **93**, 206-209 (2014).
17. Stukowski, A. Visualization and analysis of atomistic simulation data with OVITO-the Open Visualization Tool. *Modell. Simul. Mater. Sci. Eng.* **18**, 015012 (2010).
